# Structure and Genetic Variability of the Oceanic Whitetip Shark, *Carcharhinus longimanus*, Determined Using Mitochondrial DNA

**DOI:** 10.1371/journal.pone.0155623

**Published:** 2016-05-17

**Authors:** Sâmia M. Camargo, Rui Coelho, Demian Chapman, Lucy Howey-Jordan, Edward J. Brooks, Daniel Fernando, Natalia J. Mendes, Fabio H. V. Hazin, Claudio Oliveira, Miguel N. Santos, Fausto Foresti, Fernando F. Mendonça

**Affiliations:** 1 Laboratório de Biologia e Genética de Peixes, Instituto de Biociências de Botucatu, Universidade Estadual Paulista, (UNESP), Botucatu, São Paulo, Brasil; 2 Instituto Português do Mar e da Atmosfera (IPMA), Olhão, Portugal; 3 Centro de Ciências do Mar (CCMAR), Universidade Algarve, Faro, Portugal; 4 School of Marine and Atmospheric Science, Stony Brook University, Stony Brook, New York, United States of America; 5 Microwave Telemetry, Inc., Columbia, Maryland, United States of America; 6 Shark Research and Conservation Program, Cape Eleuthera Institute, Eleuthera, The Bahamas; 7 The Manta Trust, Catemwood House, Corscombe, Dorchester, United Kingdom; 8 Department of Biology and Environmental Science, Linnaeus University, Lund, Sweden; 9 Blue Resources, Colombo, Sri Lanka; 10 Departamento de Pesca e Aquicultura, Universidade Federal Rural de Pernambuco, UFRPE, Recife, Pernambuco, Brasil; 11 Laboratório de Genética Pesqueira e Conservação, Instituto do Mar, Universidade Federal de São Paulo (UNIFESP), Santos, São Paulo, Brasil; Tuscia University, ITALY

## Abstract

Information regarding population structure and genetic connectivity is an important contribution when establishing conservation strategies to manage threatened species. The oceanic whitetip shark, *Carcharhinus longimanus*, is a highly migratory, large-bodied, pelagic shark listed by the IUCN (International Union for Conservation of Nature) Red List as "vulnerable" throughout its range and “critically endangered” in the western north Atlantic. In 2014, the species was protected globally under Appendix II of CITES (Convention on International Trade in Endangered Species), limiting and regulating trade. This study used partial sequences of mitochondrial DNA (mtDNA) control region to determine the population genetic structure of oceanic whitetip sharks across the Atlantic and Indian Oceans. 724 base pairs were obtained from 215 individuals that identifed nine polymorphic sites and defined 12 distinct haplotypes. Total nucleotide diversity (π) was 0.0013 and haplotype diversity (h) was 0.5953. The Analysis of Molecular Variance (AMOVA) evidenced moderate levels of population structure (*ɸ*_ST_ = 0.1039) with restricted gene flow between the western and eastern Atlantic Ocean, and a strong relationship between the latter region and the Indian Ocean. Even though the oceanic whitetip is a highly migratory animal the results presented here show that their genetic variability is slightly below average of other pelagic sharks. Additionally, this study recommends that at least two populations in the Atlantic Ocean should be considered distinct (eastern and western Atlantic) and conservation efforts should be focused in areas with the greatest genetic diversity by environmental managers.

## Introduction

The effects of unsustainable fishing on populations of sharks and rays (elasmobranchs) has been well-documented globally [1], and studies have shown that over the last several decades many large migratory species commonly caught in large scale pelagic marine fisheries are rapidly declining [2, 3]. The *International Union for Conservation of Nature* (IUCN), which quantifies the conservation status of most taxa, currently lists over a thousand species of elasmobranchs [4, 5]. The highly migratory sharks are amongst the species with highest conservation concerns [6].

The oceanic whitetip shark, *Carcharhinus longimanus*, is a pelagic shark with worldwide distribution, found mainly in tropical and subtropical open waters [7, 8]. Historically grouped with the silky shark *C*. *falciformis* and the blue shark *Prionace glauca*, *C*. *longimanus* was once considered a highly abundant oceanic shark [7–11]. However, recent population assessments and anecdotal reports suggest that the oceanic whitetip shark is now only occasionally recorded in the Atlantic. A recent study by Coelho et al. [12] covering a wide area of the Atlantic in both hemispheres indicated that the oceanic whitetip shark bycatch in pelagic longline fisheries is less than 1% of the total elasmobranch catches. Furthermore, philopatric behavior, defined as the tendency of individuals to return or stay in their home areas, natal (birth) sites or adopted locales[13], was recently described in this species, highlighting the need for designated protective areas for oceanic whitetip sharks.

The IUCN Red List currently lists *C*. *longimanus* as "vulnerable" throughout its range and “critically endangered” in the western north Atlantic [14], and in 2014 was listed in Appendix II of CITES (Convention on International Trade in Endangered Species), regulating international trade and requirement of appropriate permits, including documentation validating that catch is legally sourced. A 2012 Pacific-wide stock assessment for oceanic whitetip sharks showed that the populations are currently overfished and stocks have declined to levels below maximum sustainable yield [15]. In the Atlantic and Indian Oceans, Ecological Risk Assessments for pelagic sharks, show that the oceanic whitetip shark has a relatively high vulnerability to pelagic fisheries, due to its low fecundity (average three pups per litter] and high susceptibility [16, 17]. Retaining oceanic whitetip sharks for commercial purposes has been prohibited in tuna RFMOs (Regional Fisheries Management Organizations) of all Oceans, including the ICCAT (International Commission for the Conservation of Atlantic Tunas) Atlantic convention area since 2010 (ICCAT Recommendation 10–07), the IOTC (Indian Ocean Tuna Commission) Indian Ocean convention area since 2013 (IOTC Resolution 13/06), the IATTC (Inter-American Tropical Tuna Commission) in the eastern Pacific since 2012 (Resolution C-11-10), and the WCPFC (Western and Central Pacific Fisheries Commission) in the western and central Pacific since 2013 (CMM 2011–04).

An aspect that is fundamental to sustainable use of marine resources and quantification of productivity of ocean ecosystems is the identification and maintenance of distinct stocks [18]. The conservation of the genetic variability is one of the basic objectives in regulatory programs assisting in the recovery of endangered species [19]. As such, the sequencing of the mitochondrial DNA control region (D-loop) is one of the most commonly applied method to population genetic studies of vertebrates, including sharks [20–26].

Population declines, such as that observed in the oceanic whitetip shark over the last 50 years, raises questions about the maintenance of genetic variability and the putative loss of evolutionary lineages. These issues are more relevant in species with population structure where the distribution of genetic characteristics is geographically limited. Therefore, this study aimed to describe the genetic variability and population structure of *C*. *longimanus* from the Atlantic and Indian Oceans by using control region of mitochondrial DNA with an intention to provide a framework for future assessments and tracking studies.

## Materials and Methods

### Sampling

Samples of *C*. *longimanus* were collected by fishery observers on Portuguese, Brazilian and American commercial pelagic longline vessels in several regions of the Atlantic and Indian Oceans. Tissue samples, including small muscle or fin fragments (<1cm^3^), were collected opportunistically during the normal fishing operations of the vessels, and were subsequently frozen in 95% ethanol. Tissue samples from the north Atlantic were collected in The Bahamas as part of an ongoing study of oceanic whitetip movement and life history. All samples were collected under the Cape Eleuthera Institute research permit (MAF/FIS/17 and MAF/FIS/34) and in accordance with Stony Brook University and Cape Eleuthera Institute regulations developed within the guidelines of the Association for the Study of Animal Behaviour and the Animal Behavior Society [27]. Sampling from the Portuguese fishery was conducted by the Portuguese Institute for the Ocean and Atmosphere (IPMA), within the scope of the European Data Collection Framework (PNAB/DCF). This sampling was conducted under an ICCAT permit for biological sampling of shark species in the Atlantic (ICCAT Recommendation 13/10) and an IOTC permit for biological sampling of oceanic whitetip sharks in the Indian Ocean (IOTC Resolution 13/06). No specific permissions were required for internationally transporting samples of *C*. *longimanus* before the species effectively became part of CITES in September 2014, all samples used in this study were collected before that date.

**Fig 1:**
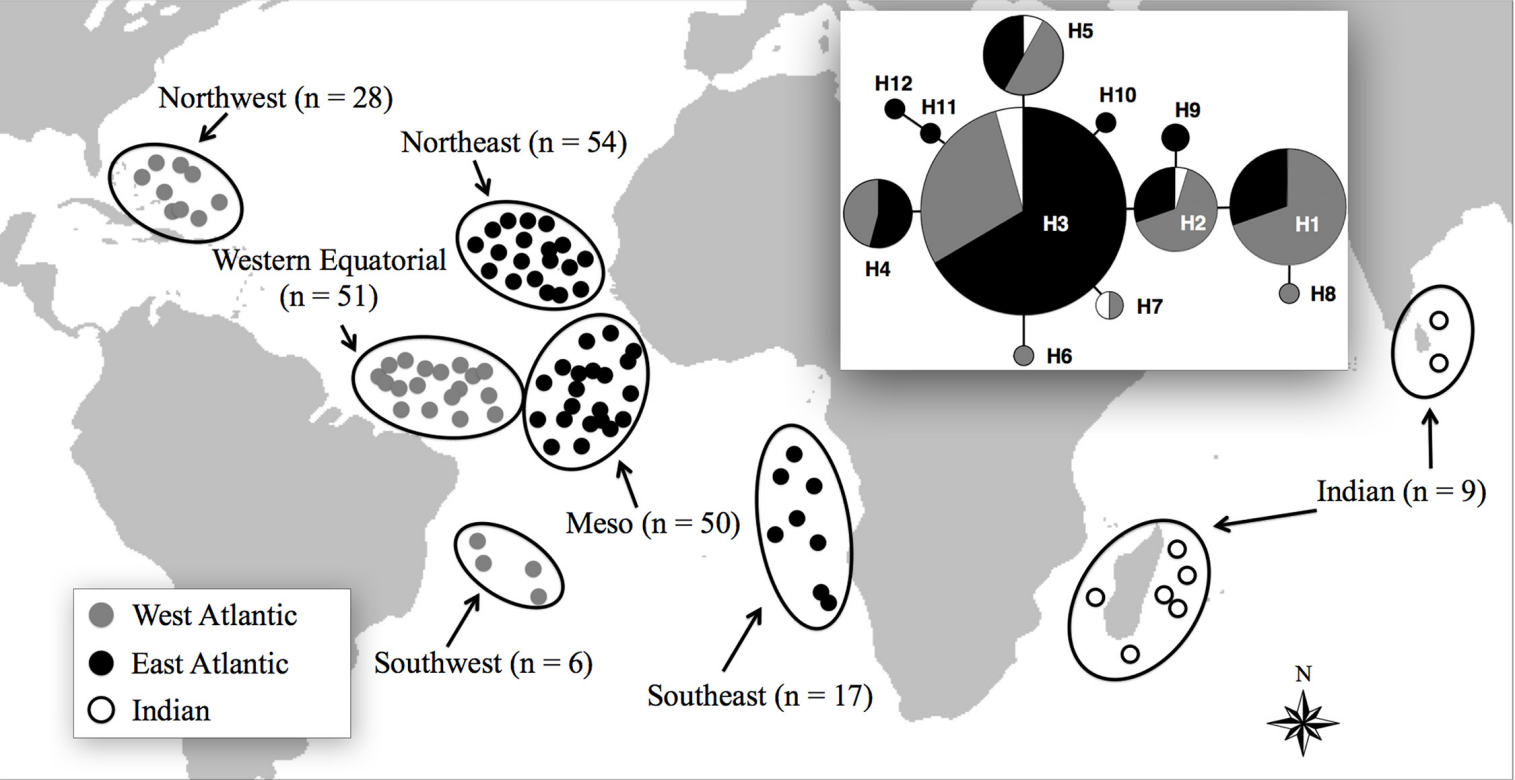
Geographic distribution of samples of *Carcharhinus longimanus* with the network haplotypes analyzed and compiled from the sequences of the mitochondrial DNA control region.

A total of 215 specimens were sampled for this study. Specifically, 206 were sampled from various regions of the Atlantic Ocean, including 28 from the Northwest Atlantic, 51 from the Western Equatorial Atlantic, 54 from the Northeast Tropical Atlantic, 50 from the Meso-Atlantic, 17 from the Southeast Atlantic and six from the Southwest Atlantic. In addition, nine samples were collected from the Indian Ocean (Fig 1).

### DNA extraction and sequencing

Genomic DNA was extracted using the NucleoSpin® Tissue XS Kit (Macherey & Nagel, Düren, Germany). Partial sequences of the control region of the mitochondrial DNA were attained according to Mendonça et al. [28]. Individual reactions were performed with approximately 30 ng DNA template, 3.2 pmol primer, 1 μl terminator mix, and 5 μl Better Buffer (The Gel Co.) in a total volume of 15 μl. PCR sequencing profiles consisted of an initial denaturation step of 4 min at 96°C, followed by 30 cycles of 30s at 96°C, 15s at 50°C, and 4 min at 60°C. Cycle sequencing was performed with BigDye Terminator v3.1 Cycle Sequencing Kit (Applied Biosystems). Sequencing was completed on an automated sequencer, ABI 3130, manufactured by Applied Biosystems. The consensus sequences were assembled and edited using Geneious 4.8.5 [29] and aligned with Muscle algorithm implemented within Geneious 4.8.5. The generated haplotypes were deposited in GenBank under accession numbers KT160318 to KT160329.

### Population genetics analyses

The relative nucleotide composition, number of polymorphic sites, haplotype diversity (*h*), nucleotide diversity (*π*), and number of pairwise nucleotide differences among populations were calculated using ARLEQUIN 3.5.1.3 software [30]. To estimate levels of genetic divergence among populations of *C*. *longimanus* index *Φ*_ST_ was calculated using the Analysis of Molecular Variance (AMOVA) [31] under the nucleotide evolution model of Tamura-Nei [32]. The *Φ*_ST_ estimates were tested with 1.000 non-parametric bootstrap pseudo-replicates using the ARLEQUIN 3.5.1.3 software, and the p-values were adjusted for simultaneous pairwise comparisons using the sequential Bonferroni correction [33]. The AMOVA test considered the possibilities of the maximum and minimum number of populations using hypothetic groups of population structure. Groupings that were most and least inclusive were tested, first by geographic proximity of the collection points, considering maritime currents; and secondly, by using the geographical distribution and correlation between the North and South, and East and West Atlantic. A minimum-spanning haplotype network was estimated using the Network 4.611 program [34]. The mismatch analysis in ARLEQUIN 3.5.1.3 included a raggedness index to determine goodness-of-fit to a unimodal distribution [35].

## Results

Among the 215 specimens of *C*. *longimanus* analyzed, we obtained a total matrix with 724 bp with 9 polymorphic sites, yielding 12 haplotypes (Table 1).

**Table 1:** Polymorphisms found in the twelve haplotypes of *Carcharhinus longimanus* along several regions of the Atlantic and Indian Oceans.

Polymorphic sites									
	1	2	2	2	2	2	3	4	5
Haplotypes	9	1	3	5	7	9	9	0	2
	2	0	0	0	6	4	9	0	9
1	G	C	T	A	T	G	T	C	T
2	.	.	.	.	.	.	C	.	.
3	.	.	C	.	.	.	C	.	.
4	.	.	C	.	.	A	C	.	.
5	.	.	C	.	C	.	C	.	.
6	.	.	C	G	.	.	C	.	.
7	.	.	C	.	.	.	C	.	C
8	.	.	.	.	C	.	.	.	.
9	.	.	.	.	.	A	C	.	.
10	A	.	C	.	.	.	C	.	.
11	.	T	C	.	.	.	C	T	.
12	.	T	C	.	C	.	.	T	.

In the overall analyses, we found low relative levels of haplotype diversity *h* = 0.5953 and nucleotide diversity *π =* 0.0013, with the greatest diversities found in the Southwest Atlantic (*h* = 0.8000, *π* = 0.00184) and in the Meso-Atlantic (*h* = 0.7571, *π* = 0.00192. Haplotype numbers three (H3 = 131 individuals) and one (H1 = 32 individuals) were the most common and were found in all sampled regions, representing 75.8% of the analyzed oceanic whitetip shark specimens. Numbers of polymorphic sites, haplotypes, nucleotide diversity and haplotypic diversity for different samples are shown in Tables 2 and 3.

**Table 2:** Geographical distribution of haplotypes of *Carcharhinus longimanus* delimited by the different areas of the Atlantic and Indian Oceans.

	West Atlantic				East Atlantic				
	Northwest Atlantic (n = 28)	West.Eq. Atlantic (n = 51)	Southwest Atlantic (n = 6)	Total (n = 85)	Meso-Atlantic (n = 50)	Northeast Atlantic (n = 54)	Southeast Atlantic (n = 17)	Indian Ocean (n = 9)	Total (n = 130)
h1	8	13	1	**22**	8	1	1	.	**10**
h2	4	7	.	**11**	3	1	1	1	**5**
h3	13	22	3	**38**	22	50	15	6	**91**
h4	1	3	1	**5**	6	.	.	.	**6**
h5	1	4	1	**6**	7	1	.	1	**9**
h6	1	.	.	**1**	.	.	.	.	.
h7	.	1	.	**1**	.	.	.	1	**1**
h8	.	1	.	**1**	.	.	.	.	**1**
h9	.	.	.	.	1	1	.	.	**2**
h10	.	.	.	.	1	.	.	.	**1**
h11	.	.	.	.	1	.	.	.	**1**
h 12	.	.	.	.	1	.	.	.	**1**

h, haplotype.

**Table 3:** Population statistics of *Carcharhinus longimanus—*n, number of individuals; P, polymorphic sites; Nh, number of haplotypes; h, haplotype diversity; π, nucleotide diversity.

	n	P	Nh	h	*π*
Northwest Atlantic	28	5	6	0.7037	0.00177
Western Equatorial Atlantic	51	5	7	0.7431	0.00170
Southwest Atlantic	6	4	4	0.8000	0.00184
**West Atlantic**	**85**	**6**	**8**	**0.716**	**0.00165**
Meso-Atlantic	50	7	9	0.7567	0.00192
Northeast Atlantic	54	4	5	0.1440	0.00034
Southeast Atlantic	17	2	3	0.2279	0.00047
Indian Ocean	9	3	4	0.5833	0.00092
**East Atlantic**	**130**	**8**	**10**	**0.471**	**0.00104**

The AMOVA test was adopted following hypothetic groups of population structure. While examining different scenarios of structuring, a greater difference (*ɸ*_ST_ = 0.1039, *P* <0.001) was observed when populations were grouped in two geographic regions: Eastern (Northeast Atlantic, Southeast Atlantic and Meso-Atlantic) and Western (Northwest Atlantic, Western Equatorial Atlantic and Southwest Atlantic) Atlantic Ocean. In this case, the indices related to genetic variability were *h* = 0.716 and π = 0.00165 in West Atlantic, and *h* = 0.471 and π = 0.00104 in the East Atlantic. Due to the low sampling rate in the the Indian Ocean, the AMOVA did not detect a statistically significant structure when left as a distinct group. However, in the *F*_ST_ pairwise analysis an absence of structure was observed between the Indian Ocean and the East Atlantic groups (Table 4).

**Table 4:** Differentiation index (*F*_ST_) between pairs of sampled regions in the Atlantic and Indian Oceans. Pairwise *F*_ST_ numbers are below the diagonal line, and the significance of the p-values are represented above the diagonal (+ represents a statistically significant difference with p-value < 0.01).

	Northwest Atlantic	Western Eq.Atlantic	Southwest Atlantic	Mezo- Atlantic	Northeast Atlantic	Southeast Atlantic	Indian Ocean
Northwest Atlantic	0	-	-	-	+	+	-
Western Eq. Atlantic	- 0.0228	0	-	-	+	+	-
Southwest Atlantic	- 0.0005	-0.0224	0	-	-	-	-
Mezo- Atlantic	0.0309	0.0201	-0.0943	0	+	-	-
Northeast Atlantic	0.2716	0.2085	0.1352	0.0965	0	-	-
Southeast Atlantic	0.1170	0.0949	0.0081	0.0290	-0.0137	0	-
Indian Ocean	0.1092	0.0814	-0.0623	0.0019	0.0237	-0.0224	0

A statistical parsimony haplotype network was constructed using the Network 4.611 program. More than 60% analyzed sequences shared a single haplotype (H3), which was found in all sampled regions (Fig 1).

## Discussion

### Population genetics of the oceanic whitetip shark

The results show low levels of genetic diversity for the oceanic whitetip shark in the Atlantic and also in the Indian Ocean, although the latter has been evaluated with a small number of individuals and in a relatively small area. Population genetic studies with marine vertebrates, such as bony fishes, cetaceans and other elasmobranchs (including pelagic sharks) also tend to report low values of nucleotide and haplotypic diversity in the Atlantic Ocean [36–39]. Although low levels of nucleotide diversity are not considered standard for elasmobranchs, similar results to those obtained for oceanic whitetip sharks have been found in other highly migratory pelagic species including basking shark (*Cetorhinus maximus)*, crocodile shark (*Pseudocarcharias kamoharai)*, the whale shark (*Rhincodon typus)*, pelagic thresher shark (*Alopias pelagicus)* and the white shark (*Carcharodon carcharias)* [25, 26, 38, 40–44]. The combination of low haplotypic diversity with a single haplotype shared by most of the individuals is also found in the nurse shark (*Ginglymostoma cirratum)* [39], white shark (*Carcharodon carcharias)* [43] and sicklefin lemon shark (*Negaprion acutidens)* [44].

Considering two distinct groups for the oceanic whitetip shark in the Atlantic Ocean and based on the hypothetic population groupings and genetic structuring simulations, restrictions to gene flow with moderate genetic divergence were observed, with one population occurring in the West and another in the East Atlantic. Population structure between the eastern and western portions of an oceanic basin was observed by Cardeñosa et al [41] for the thresher shark with strong differentiation in the Pacific. In the latest study on population genetic structure of the white shark, presented by O'Leary et al. [43], a similar structure was observed to the one in our study, being the *Φ*_ST_ = 0.10 with sequences of mtDNA control region and *F*_ST_ = 0.1057 with microsatellite marker, between the northwest Atlantic and southern Africa.

It would not be surprising to observe panmitic populations given the migration potential of this species. However, ocean currents and their interactions produce the oceanic barriers, organismal dispersal potential, behavior and organismal environmental tolerances which all contribute to patterns of diversification in the oceans [45, 46]. Scientific findings indicate that the western Atlantic is an area of both origin and accumulation of biodiversity [47]. This is supported by population growth rates of *C*. *longimanus* as rates of haplotype and nucleotide diversity are approximately 35% higher in western Atlantic. The haplotype network in star contraction generated from the haplotype H3 suggests an expansion event, possibly from the individuals of the eastern Atlantic, where the highest rates were observed in diversity with higher frequency on the H3.

Currently, very little is known about the movements and habitat use of oceanic whitetips in the Atlantic. The most comprehensive movement study of this species used Pop-up satellite archival tags applied to 11 mature females and one mature male,.near Cat Island in the central Bahamas. In that study, the maximum individual displacement from the tagging site dispersed 290–1940 km after 30–245 days at liberty, with individuals moving to several different destinations. The tag affixed to the male shark never reported. All of the tagged oceanic whitetips remained within 500 km of their tagging location for the first 30 days of their tracks and none of them moved away from the northwestern Atlantic [27].

The shortest route between the western and eastern Atlantic is between Brazil and Guinea-Bissau, requiring an oceanic crossing measuring ~2400 km. Although this distance does not seem to be a barrier to potential oceanic whitetip migration, such a trans-Atlantic route does not seem to be an aspect of the female behavior of the species. In those regions the oceanic whitetip shark populations in the western and east Atlantic are genetically differentiated from one another at mitochondrial loci, at least by female lineages. Philopatry is one of the factors that could also influence the reduction of gene flow across the Atlantic, by the migration of females to their original birth place [27]. Philopatry is well documented in many other sharks reviewed in Chapman et al. [48] and trans-Atlantic structure may have developed in oceanic whitetips because females remain within or return to give birth on one side of the basin or the other. A survey of biparentally inherited genetic markers is needed to determine whether or not there is male-mediated gene flow across the Atlantic.

The haplotype network denotes genetic differences among individuals without showing correlations between the different branches and their geographic distributions, with a characteristic pattern of an unstructured population. Although dispersion of fishes between the Indian and Atlantic Oceans are rarely observed, mtDNA data show that some species of tropical and sub-tropical fish found in the Indian Ocean may cross the barrier of the Benguela current [49]. The water transport between the Indian and Atlantic Oceans is summarized in [50]. A strong genetic connectivity between the Indian Ocean group and the Eastern Atlantic groups through the pairwise *F*_ST_, despite low statistical significance, highlight the dispersal capability of *C*. *longmanus*. Considering the movement of individuals between Indian and eastern Atlantic, the absence of significant genetic structure can indicate the existence of only one genetic stock of oceanic whitetip sharks around the African continent, both in the eastern Atlantic and western Indian Ocean. The warm Agulhas current passes the Cape of Good Hope and is incorporated into the cool Benguela current. The Agulhas current is one of the strongest currents in the world, and it may help the transport of oceanic whitetip sharks from the southwest Indian Ocean to the Southeast Atlantic. However, the question on whether the flow of these sharks can also occur in the reverse order remains unknown.

More genetic studies and their importance in the evolutionary history of elasmobranchs with oceanic distribution, as well as for the management of commercially exploited shark species, are needed. Phylogeographic studies presenting extensive sampling and appropriate geographic scales including habitats with different ecological characteristics (such as oceanic and continental, tropical or subtropical) have a greater chance of detecting evolutionarily significant units, which would facilitate the development and application of appropriate conservation measures [51].

### Conservation of the oceanic whitetip shark

Though shark populations are often impacted by overfishing, pelagic species, including *C*. *longimanus*, ubiquitous in all tropical oceans, with its susceptibility to incidental longline capture, make it prone to population declines, prompting the IUCN to designate it “vulnerable”. Considering the important relationship between the sustainability of a natural population and their genetic diversity, there is an urgent need to uncover information about global levels of genetic diversity and geographical distribution as well as patterns of gene flow and the detection of significant evolutionary units.

Here we present evidence that two populations of oceanic whitetip sharks exist in the Atlantic, specifically one in the western and another in the eastern regions of the basin. Despite their highly migratory nature, barriers to the gene flow of oceanic whitetips in the Atlantic result in two genetically distinct and demographically independent populations. This structure should be incorporated into assessments and monitoring of this species. The factors that restrict gene flow in the Atlantic may be absent between the east Atlantic and parts of the Indian Ocean; as there appears to be connectivity between those two regions. Isolated populations, especially if reduced by fishing or other sources of mortality, are vulnerable to inbreeding as reproducing adults have an elevated probability of mating with relatives [52]. Nonrandom matings generate deviations in allele frequencies with heterozygous deficiencies. Therefore, resource managers, tasked with the conservation of this species, should recognize that intrinsic reproductive barriers need to be considered concurrently with immigration rates, for sustaining at-risk populations.In addition to the low levels of genetic variability found for whitetip sharks throughout the study area, an important difference was observed between the two populations, with the haplotype diversity of the eastern population 34.2% less than the western population. This difference was even more striking for the nucleotide diversity with 36.9% less in the eastern population. These low genetic variability rates found may represent a dramatic risk to the adaptive potential of the species leading to a weaker ability to respond to environmental changes, and consequently, could promote extinction of some lineages in the future. Describing differences in indices of genetic diversity allows for managers to prioritize conservation efforts between populations with the highest diversity index, maintaining evolutionary potential, or populations with the lowest genetic variability, in an effort to prevent further inbreeding and reduced reproductive success. In order to prevent further population declines, we suggest that global genetic diversity should be preserved for all haplotypes through multinational cooperation, and particularly for the two distinct populations of oceanic whitetips identified in the Atlantic.
